# Antibacterial, antibiofilm and anti-quorum sensing activities of 1,2,3,5-tetrazine derivatives linked to a benzothiazole moiety

**DOI:** 10.1371/journal.pone.0318135

**Published:** 2025-06-03

**Authors:** Jean Paul Dzoyem, Joseph Tsemeugne, Boniface Pone Kamdem, Rostand Foyou Meupiap, Boris Arnaud Kuate, Pierre Mkounga, Fabrice Fekam Boyom, Lyndy Joy McGaw

**Affiliations:** 1 Department of Biochemistry, Faculty of Science, University of Dschang, Dschang, Cameroon; 2 Phytomedicine Programme, Department of Paraclinical Sciences, University of Pretoria, Pretoria, South Africa; 3 Laboratory of Natural Products and Applied Organic Synthesis (LANAPOS), Department of Organic Chemistry, Faculty of Science, University of Yaounde I, Yaounde, Republic of Cameroon; 4 Antimicrobial and Biocontrol Agents Unit (AmBcAU), Laboratory for Phytobiochemistry and Medicinal Plants Studies, Department of Biochemistry, Faculty of Science, University of Yaounde I, Yaounde, Cameroon; 5 Advanced Research & Health Innovation Hub, Yaounde, Cameroon; Universidad de Valparaiso, CHILE

## Abstract

A series of known tetrazine derivatives, containing benzothiazole scaffold, were prepared during the coupling reactions of selected diazotized 2-aminobenzo[d]thiazole derivatives with p-acetaminophen. The as-prepared compounds were characterized based on NMR and MS spectrometry. The antibacterial and anti-biofilm activities of the synthesized compounds were evaluated by microdilution method, whereas the anti-quorum sensing effect was carried out using assay for the inhibition of violacein formation. As a result, compounds **4a**, **4b** and **4c** revealed minimum inhibitory concentrations and minimum bactericidal concentrations ranging from 8 to 128 µg/mL and from 32 to 256 µg/mL, respectively. Compounds **4a** (52–86.5%), **4b** (57.7–79.4%) and **4c** (59.9–80.3%) prevented biofilm formation in all the four bacteria tested with percentages of inhibition more than 50%. The concentrations of **4a**, **4b** and **4c** that inhibited 50% of violacein production were found to be 62.71, 28.56 and 107.45 µg/mL, respectively, thus attesting that these compounds possess anti-quorum sensing activity. Noteworthy, our previous investigation attested that these compounds are non-cytotoxic on the human mammalian cells Vero. This novel contribution demonstrates the antibacterial, antibiofilm and anti-quorum sensing activities of tetrazine-based benzothiazoles, which might be prospected as scaffolds for the discovery of efficient antibiotics with decreased risk of microbial drug resistance.

## Introduction

Resistance to currently available antibiotics has become a grave menace to the treatment of infectious diseases [1,2]. According to the 2019’s World Health Organization (WHO) report, antibacterial resistance was accountable for 1.27 million deaths worldwide [3,4]. The main mechanisms of this notorious phenomenon include enzymatic alteration and efflux pump systemic elimination of antimicrobials, structural modification of bacterial target proteins, changes in bacterial membrane permeability, etc. In the meantime, many pathogenic bacteria can form a dense biofilm [5,6], thus making bacteria highly resistant to antibiotics [7]. In fact, the contribution of biofilms in antimicrobial resistance is highly complex and may significantly drive resistance [8–10]. By controlling the formation of biofilms and drug efflux pumps, the quorum sensing system plays a crucial role in developing bacterial drug-resistant pathways [11]. Growing evidence has demonstrated the link between quorum sensing and biofilm development [12–14]. As a matter of fact, a quorum sensing is a density-dependent cell-signalling mechanism by which bacteria crosstalk to each other [14,15]. Because of its connection to bacterial pathogenicity, virulence and biofilm formation, quorum sensing has gained more research attention in the last decade [16,17]. Thus, targeting quorum sensing and biofilm formation would be a prominent approach to unravel antibiotic resistance in pathogenic bacteria [18,19].

Modern research has substantially identified antibacterial compounds with anti-quorum sensing and anti-biofilm properties [6,20–24], even though almost no such chemotype is reported to have succeeded the last clinical trial stage in humans.

Thus, the development of effective chemotypes that inhibit bacterial growth while preventing biofilm formation and attenuating quorum sensing-dependent virulence factors is of paramount importance.

Benzothiazole is a heterocyclic and bicyclic pharmacophore that contains benzene fused with 1,3-thiazole skeleton [25,26]. A number of scientists have established the potential of benzothiazole and its derivatives as antimicrobial hit compounds [27–29].

On the other hand, modern research on tetrazine derivatives in relation to their antibacterial activity has been reported by many researchers [30,31].

The incorporation of 1,2,3,5-tetrazine derivatives into the benzothiazole structure introduces new chemical functionalities that can potentially disrupt bacterial biofilms and interfere with quorum sensing mechanisms. This dual approach aims not only to inhibit bacterial growth but also to prevent biofilm formation and attenuate virulence factors, thereby offering a comprehensive strategy to combat bacterial infections.

In our previous research investigation, we demonstrated the cytotoxic effects of 1,2,3,5-tetrazine tethered benzothiazole derivatives against a number of cancer cells, including A549, Hela and MCF-7 cells. However; these compounds were non cytotoxic vis-à-vis the human mammalian cells Vero.

In our continuing effort to search for effective antibacterial hit compounds that might aid in drug discovery campaigns, this study sought to investigate the inhibitory effects of certain 1,2,3,5-tetrazine tethered benzothiazole derivatives on the growth of selected bacteria. Moreover, anti-biofilm and anti-quorum sensing activities of these compounds are also investigated.

## Materials and methods

### Chemistry

#### General.

The reagents of analytical grade were purchased from commercial sources and used without any further purification. ^1^HNMR spectra were measured with a 400 MHz spectrometer NMR Bruker Advance 400 at room temperature in DMSO-d_6_ with tetramethylsilane as the internal reference. ^13^C-NMR spectra were recorded in DMSO-d_6_ with a 100 MHz spectrometer NMR Bruker Advance 400. UV-visible absorption spectra were recorded on Beckman U-640 Spectrophotometer, using samples’ solutions of concentration 5 × 10^−5^ mol.L^−1^. Infrared spectra were taken in KBr on a Perkin Elmer FT-IR 2000 spectrophotometer. Masss spectra were measured with a Waters Xevo TQD tandem quadrupole mass spectrometry system running in MS scan mode, 1 minute of acquired spectra were combined and centroided. Melting points were obtained with a Buchii melting point apparatus and are uncorrected. The Thin Layer Chromatography (T.L.C.) was carried out on Eastman Chromatogram Silica Gel Sheets (13181; 6060) with fluorescent indicators. A mixture of hexane and ethyl acetate (4:6) was used as the eluent and iodine was used for the visualization of the chromatograms.

#### Preparation of diazonium salt solution.

As per a previously reported protocol [32], dried sodium nitrite (0.69 g, 10 mmol) was slowly added over a period of 30 minutes to concentrated sulphuric acid (10 mL) with occasional stirring. The solution was cooled to 0–5 °C. Compound **1** was dissolved in DMSO (10 mL) and cooled to 0–5 °C. The nitrosyl sulphuric acid solution was added to the solution of **1** and the temperature was maintained between 0–5 °C. The clear diazonium salt solution thus obtained consisting of the *in situ*-formed intermediate **2**, was used immediately in the coupling reactions.

#### General procedure for the preparation of the coupling products (4).

Acetaminophen (**3**) (1.51 g, 10 mmol) or 2-amino-6-nitrobenzothiazole (**1b**) (1.952 g, 10 mmol) was dissolved in DMSO (10 mL) and then cooled in an ice-bath at 0–5 °C. A prepared diazonium solution of **2** was added drop wise over 1 hour, and then 15 mL of sodium acetate solution (10%) was added to the mixture. The pH of the mixtures was in the range 9–11. The solid precipitate was collected on a filter and crystallised from methanol to give the title compound.

#### N-(3-((5,6-dimethylbenzo[d]thiazol-2-yl)diazenyl)-4-hydroxyphenyl)acetamide (4a) .

Compound **4a** was obtained in 58% yield as red powder; m.p. 118–120 °C; [Litt: 119–121 °C, [32]; ^1^H-NMR (DMSO-d_6_, 400MHz): δ 10.67 (s, 1H, O-H), 9.94 (s, 1H, N-H), 8.10 (d, 1H, J = 4.0 Hz, H-2’), 7.89 (s, 1H, H-4), 7.84 (s, 1H, H-7), 7.60 (dd, 1H, J = 4.0 and 8.0 Hz, H-6’), 7.06 (d, 1H, J = 8.0 Hz H-5’), 2.36 (s, 6H, 2CH_3_), 2.01 (s, 3H, COCH_3_); ^13^C-NMR (DMSO-d_6_,100 MHz): δ 174.6 (CO), 168.1 (C-2), 153.6 (C-3a), 131.3 (C-4a), 124.2 (C-4), 137.4 (C-5), 132.1 (C-6), 122.5 (C-7), 138.5 (C-1’), 108.1 (C-2’), 136.1 (C-3’), 151.1 (C-4’), 118.8 (C-5’), 128.1 (C-6’), 23.8 (CH_3_CO), 19.9 (CH_3_), 19.6 (CH_3_); UV-Vis λ_max_ (DMSO) (Log ε): 274 (5.00), 327 (4.39), 364 (4.49), 452 (4.01) nm; IR (KBr) υ_max_: 3248 (O-H and N-H), 1659 (C = O), 1604−1557 (C = C), 1483−1450 (N = N), 1274 (C-S), 1239 (C-S), 861−510 (Ar def C = N str thiazole) cm^-1^. (ESI+ ) *m/z* (%) 394 (8), 389 (10), 375 (13), 372 (7), 316 (65), 304 (48), 283 (19), 202 (14), 192 (21), 150 (70); Anal. Calcd. for C_17_H_22_N_4_O_5_S: C, 59.98; H, 4.74; N, 16.46; S, 9.42. Found: C, 59.63; H, 4.80; N, 16.41; S, 9.40. Rf = 0.62.

#### 4-((5-acetamido-2-hydroxyphenyl)diazenyl)-3-(2-mercapto-4,5-dimethylphenyl)-7,8-dimethylbenzo[4,5]thiazolo[2,3-d][1,2,3,5]tetrazine-3,5-diium sulfate (4a’).

Compound **4a’** was obtained in 34% yield as brown powder; m.p. 318–319 °C; [Litt. 318–320 °C [32]; ^1^H-NMR (DMSO-*d*_*6*_, 400 MHz): δ 11.35 (s, 1H, OH), 10.67 (s, 1H, NH), 8.37 (s, 1H, H-9”’), 8.13 (d, 1H, J = 2.8 Hz, H-6’), 7.93 (s, 1H, H-6”’), 7.77 (s, 1H, H-6”), 7.69 (s, 1H, H-3”), 7.63 (dd, 1H, J = 8.8 and 2.8 Hz, H-4’), 7.09 (d, 1H, J = 8.8 Hz, H-3’), 2.50, 2.39, 2.37, 1.23 (s, 12H, CH_3_), 2.04 (s, 3H, COCH_3_); ^13^C-NMR (DMSO-*d*_*6*_, 100 MHz): δ 198.6 (C-4), 174.5 (C = O), 168.1 (C-6), 153.5 (C-1”), 151.0 (C-2’), 138.4 (C-5”’), 137.4 (C-8”’ and C-5’), 136.0 (C-5”), 132.1 (C-7”’), 131.3 (C-4”’ and C-2”), 128.1 (C-1’), 124.2 (C-4”), 122.4 (C-3”), 122.1 (C-9”’), 121.6 (C-6”’), 118.8 (C-6”), 118.3 (C-4’), 108.0 (C-3’), 105.4 (C-6’), 23.8 (COCH_3_), 23.7, 19.86, 19.7, 19.6 (Ph-CH_3_); UV-Vis λ_max_ (MeOH) (Log ε): 227 (4.06), 257 (4.12), 272 (4.26), 290 (4.09), 295 (4.08), 302 (4.12), 325 (4.19), 348 (4.18), 355 (4.19), 399 (4.23), 445 (4.25), 486 (4.22) nm; IR (KBr) ν_max_: 3887−3282 (O-H and N-H), 2920 (ArC-H), 2324 (S-H), 1664−1655 (C = O), 1533 (C = C), 1490−1449 (N = N), 1370 (δ_tetrazine ring_), 1269 (C-S), 1240 (C-O), 889 (δ_tetrazine ring_) cm^-1^; ms: (ESI+ ) *m/z* (%) 699 (8), 673 (9), 643 (11), 659 (75), 601 (10), 599 (58), 581 (22), 485 (41), 410 (34), 409 (74), 316 (100), 166 (47); Anal. Calcd. for C_26_H_33_N_7_O_10_S_3_: C, 44.63; H, 4.75; N, 14.01; S, 13.74. Found: C, 44.59; H, 4.80; N, 14.05; S, 13.71. Rf = 0.30.

#### N-4-Hydroxy-2,3-bis[3-(3-Nitro-benzenethiol-5)-yl-7-nitro-9-thia-1,2-diaza-3,4a-diazonia-fluorene-4)-yl-diazenyl]-5,6-bis[(6-Nitro-benzothiazol-2)-yl-diazenyl]-phenyl-acetamide disulfate (4b).

Compound **4b** was obtained in 67% yield as brown powder; m.p. 169–172 °C; [Litt. 171–173 °C [32]]; ^1^H-NMR (DMSO-*d*_*6*_, 400 MHz): δ 8.65 (d, 1H, J = 2.4 Hz, H-8^v^), 8.58 (d, 1H, J = 2.8 Hz, H-4’), 8.43 (dd, 1H, J = 8.8 and 2.8 Hz, H-5’), 8.43 (dd, 1H, J = 9.2 and 2.4 Hz, H-6^v^), 8.38 (dd, 1H, J = 8.8 and 2.4 Hz, H-4^iv^), 8.38 (dd, 1H, J = 9.2 and 2.0 Hz, H-6^vii^), 8.31 (d, 1H, J = 9.2 Hz, H-5^v^), 8.20 (dd, 1H, J = 6.4 and 2.0 Hz, H-4^viii^), 8.19 (d, 1H, J = 2.4 Hz, H-2^iv^), 8.18 (d, 1H, J = 6.4 Hz, H-2^viii^), 8.10 (dd, 1H, J = 8.8 and 2.4 Hz, H-5”), 7.85 (d, 1H, J = 8.8 Hz, H-4”), 7.68 (d, 1H, J = 2.0 Hz, H-5^viii^), 7.42 (d, 1H, J = 9.2 Hz, H-8^vii^), 7.35 (d, 1H, J = 2.0 Hz, H-5^vii^), 7.28 (d, 1H, J = 8.8 Hz, H-7’), 7.13 (d, 1H, J = 2.4 Hz, H-7”), 7.05 (d, 1H, J = 8.8 Hz, H-5^iv^), 3.17 (s, 2H, SH), 2.05 (s, 3H, COCH_3_); ^13^C-NMR (DMSO-*d*_*6*_, 100 MHz): δ 180.4 (CO), 171.7 (C-4”’), 170.5 (C-4^vi^), 169.8 (C-2’), 168.3 (C-2”), 168.0 (C-4), 158.2 (C-3^vii^), 155.8 (C-3a’), 155.3 (C-3a”), 155.1 (C-3^iv^), 152.5 (C-7^v^), 145.3 (C-6^iv^), 143.7 (C-6^vii^), 143.2 (C-7^viii^), 142.4 (C-6’), 141.9 (C-6”), 140.7 (C-9a”’ and 9a^vi^), 138.6 (C-1^vii^), 135.6 (C-7a”), 134.4 (C-7a’), 132.2 (C-5a^v^ and 5a^viii^), 131.7 (C-1^iv^), 131.4 (C-1), 129.9 (C-8a^viii^), 125.0 (C-8a^v^), 124.5 (C-6^viii^), 124.4 (C-6^v^), 122.7 (C-5^iv^), 122.3 (C-5^vii^), 122.1 (C-5^v^), 121.9 (C-5^viii^), 121.8 (C-8^viii^), 121.8 (C-4^iv^), 121.5 (C-8^v^), 120.4 (C-4^vii^), 119.8 (C-4’), 119.1 (C-4”), 119.0 (C-2), 119.0 (C-2^vii^), 118.6 (C-6), 118.4 (C-5’), 118.2 (C-5”), 117.6 (C-2^iv^), 116.8 (C-7”), 116.7 (C-7’), 111.4 (C-5), 107.1 (C-3), 23.8 (COCH_3_); UV-Vis λ_max_ (MeOH) (Log ε): 272 (4.67), 348 (4.87), 437 (4.25), 483 (4.25), 555 (3.75) nm; IR (KBr) ν_max_: 3285 (O-H and N-H), 3097 (ArC-H), 1654 (C = O), 1599 (C = N), 1512 (C = C), 1442 (N = N), 1269 (C-S), 1234 (C-O), 910−502 (Ar def C = N str thiazole) cm^-1^; ms: (ESI^+^) *m/z* (%) 1052 (4), 996 (3), 882 (3), 713 (3), 694 (3), 659 (3), 637 (7), 599 (13), 409 (16), 317 (26), 316 (81); Anal. Calcd. for C_50_H_33_N_25_O_26_S8: C, 36.26; H, 2.01; N, 21.14; S, 15.48. Found: C, 36.24; H, 1.98; N, 21.17; S, 15.43. Rf = 0.53.

#### 3,11-dinitrobenzo[4,5]thiazolo[3,2-c]benzo[4,5]thiazolo[3,2-e][1,2,3,5]tetrazine-8,14-diium sulfate (4c).

Compound **4c** was obtained in 41% yield as orange powder; m.p. 244–246 °C; [Litt. 245–247 °C [32]]; ^1^H-NMR (DMSO-*d*_*6*_, 600 MHz): δ 8.95 (d, 1H, J = 1.8 Hz, H-7), 8.69 (d, 1H, J = 2.4 Hz, H-7’), 8.29 (s, 2H, NH), 8.25 (dd, 1H, J* *= 2.8 and 8.8 Hz, H-5), 8.11 (dd, 1H, J = 2.4 and 8.8 Hz, H-5’), 7.90 (d, 1H, J = 8.8 Hz, H-4), 7.42 (d, 1H, J* *= 8.8 Hz, H-4’); ^13^C-NMR (DMSO-*d*_*6*_, 150 MHz): δ 153.2 (C-2), 155.0 (C-3a), 121.0 (C-4), 122.5 (C-5 and C-5’), 144.0 (C-6), 119.3 (C-7), 132.6 (C-7a), 172.3 (C-2’), 158.7 (C-3a’), 117.3 (C-4’), 141.2 (C-6’), 118.3 (C-7’), 131.9 (C-7a’); **UV-Vis (MeOH)** λ_max_ (log *ε*): 260 (4.54), 285 (4.48), 352 (4.66), 393 (4.62), 421 (4.63), 450 (4.64), 472 (4.62); **IR (KBr)** υ_max_/cm^-1^: 3097 (C_Ar_-H), 1556 (C = N), 1444 (N = N), 1336 (C_Ar_-NO_2_), 1120 (C-S), 1514 (C = C); ms: (ESI^+^) *m/z* (%) 590 (5), 568 (3), 562 (10), 558 (15), 554 (10), 550 (17), 514 (35), 450 (76), 378 (10), 294 (13), 248 (34). Anal. Calcd. for: C_14_H_18_N_6_O_14_S_3_: C, 28.48; H, 3.07; N, 14.23; S, 16.29. Found: C, 28.50; H, 3.1; N, 14.25; S, 16.32. Rf = 0.45.

#### 1,2-bis(6-nitrobenzothiazol-2-yl)diazene-1,2-diium sulfate (4c’).

Compound **4c’** was obtained in 28% yield as red powder; m.p. 243–245 °C; [Litt. 243–245 °C [32]; ^1^H-NMR (DMSO-*d*_*6*_, 600 MHz): δ 8.69 (d, 2H, J* *= 2.4 Hz, H-7 and H-7’), 8.24 (2H, *s*, NH), 8.10 (dd, 2H, J* *= 2.4 and 8.8 Hz, H-5 and H-5’), 7.42 (d, 1H, J* *= 8.8 Hz, H-4 and H-4’); ^13^C-NMR (DMSO-*d*_*6*_, 150 MHz): δ 172.3 (C-2 and C-2’), 159.1 (C-3a and C-3a’), 117.3 (C-4 and C-4’), 122.5 (C-5 and C-5’), 141.2 (C-6 and C-6’), 118.2 (C-7 and C-7’), 132.1 (C-7a and C-7a’). **UV-Vis (MeOH)** λ_max_ (log *ε*): 269.7 (4.53), 279.6 (4.30), 351.3 (5.17); **IR (KBr)** υ_max_/cm^-1^: 3508 (N-H), 3068 (C_Ar_-H), 1644 (C = N), 1568 (C = C), 1486 (N = N), 1282 (C_Ar_-NO_2_), 1120 (C-S); ms: (ESI^+^) *m/z* (%) 484 (5), 452 (100), 456 (5), 466 (8), 438 (10), 428 (17). Anal. Calcd. for C_14_H_8_N_6_O_8_S3: C, 34.71; H, 1.66; N, 17.35; S, 19.85 Found: C, 34.73; H, 1.70; N, 17.33; S, 19.88. Rf = 0.48.

### Antimicrobial susceptibility and antibiofilm assays

#### Microbial strains.

Strains of *Klebsiella aerogenes* ATCC 130148*, Acinetobacter baumannii* ATCC BAA-1605, *Enterococcus faecium* ATCC700221, *Staphylococcus epidermidis* ATCC35984 and *Chromobacterium violaceum* ATCC12472 from the American Type Culture Collection (ATCC) were used. They were maintained in Muller Hinton agar (MHA) at 37°C, while *Chromobacterium violaceum* strain was maintained in Luria–Bertani (LB) agar at 25°C.

#### Determination of the minimum inhibitory concentration (MIC) and the minimum bactericidal concentration (MBC).

MIC and MBC values were determined by the broth microdilution method using Muller Hinton broth (MHB). Stock solutions of compounds and reference antibacterial gentamicin were prepared in 100% dimethyl sulfoxide (DMSO; Sigma), and twofold serial dilutions were prepared in media in amounts of 100 μL per well in a 96-well plate. Then, 100 μL of a bacterial suspension was added to each well of the plate except those of the sterility control, resulting in a final inoculum of 1.5 × 10^6^ CFU/mL. The final concentration of samples ranged 0.125 to 256 μg/mL. The final concentration of DMSO was lower than 2.5% and does not affect the bacterial growth. The medium without the agents was used as a growth control, and the blank control contained only the medium. Gentamicin (final concentrations’ range: 0.031–64 μg/mL) was used as a positive control. The microtitre plates were incubated at 37°C for 24 h. The assay was repeated three times in triplicate. The MIC of the samples was detected following the addition (40 µL) of 0.2 mg/mL *p*-iodonitrotetrazolium chloride and incubation at 37°C for 30 minutes. Viable microorganisms reduced the yellow dye to a pink color. MIC was defined as the lowest sample concentration that prevented this change and exhibited complete inhibition of bacterial growth.

The MBC was determined by adding 50 μL of the suspensions from the wells, which did not show any growth after incubation during MIC assays, to 150 μL of fresh MHB. These suspensions were incubated at 37°C for 48 h. The MBC was determined as the lowest concentration of sample that inhibited bacterial viability.

#### Determination of the minimum biofilm inhibitory concentration (MBIC_50_) and the minimum biofilm eradicating concentration (MBEC_50_).

In a preliminary experiment, all the test compounds were evaluated at their MIC concentration, and those with more than 50% biofilm inhibition/eradication were selected for a dose‒response assay to determine the MBIC_50_ and MBEC_50_ values. This test was carried out by the broth microdilution method as previously described [33]. Briefly, 100 μL of MHB supplemented with 2% glucose containing the samples was introduced into the first wells followed by a serial twofold dilution. Subsequently, 100 μL of bacterial suspension was added to all wells except those of the sterility control, resulting in a final inoculum of 1.5 × 10^6^ CFU/mL, followed by incubation at 37°C for 24 h. After incubation, the plate was washed three times with phosphate-buffered saline (PBS; pH 7.2) to remove non-adherent bacteria cells. Wells containing MHB without bacteria served as the negative control. The remaining bacterial cells that attached to the well surface were considered as true biofilm. Then, the plates were stained with 0.1% crystal violet solution for 20 min at room temperature. After staining, the plates were washed three times with PBS. Then, the plates were air-dried and destained with 150 μL of 95% ethanol (v/v) for 30 min. Finally, the optical density was measured at 590 nm using a microplate reader (BioTek Epoch Microplate Spectrophotometer). Untreated wells and wells containing broth only were used as positive and blank controls, respectively, and the percentage of biofilm inhibition was calculated by using the following formula:


$$\%\;inhibition\;=\;100\;-\;[({OD}_{sample\;}-\;{OD}_{blank\;})/({OD}_{control\;}-\;{OD}_{blank\;})\;\times \;100]\mathrm{\backslash newsavebox}\mathrm{\backslash eqbox}$$

The MBEC_50_ was determined under the same conditions as the MBIC_50,_ with the only difference being that the biofilm was allowed to form for 24 h before treatment with the samples.

Median MBIC_50_ and MBEC_50_ values were defined as the concentration inhibiting 50% of biofilm formation and preformed biofilm, respectively. This was calculated by plotting the percentage of inhibition or eradication versus the concentrations using GraphPad Prism software. Samples were tested in triplicate, and experiments were repeated three times.

### Anti-quorum sensing assay

#### Inhibition of violacein production.

The inhibition of violacein production was performed according to a previously described method [34] and miniaturized in a 48-well microplate. This was achieved by transferring 1000 μL of test compounds’ solutions into the first well of a 48-well microplate, followed by a serial twofold dilution in LB broth. Then, 500 μL of *C. violaceum* inoculum, standardized at 3x10^6^ CFU/mL, was added to all wells except those of the sterility control to obtain a final concentration of 4–256 μg/mL. Vanillin, at final concentrations ranging from 0.5 to 1024 μg/mL, was used as a reference compound [35]. Plates were properly sealed with parafilm and incubated in an orbital shaker (140 rpm) at 30°C for 24 h. The MIC was defined as the minimum concentration inhibiting visible bacterial growth and therefore preventing the production of purple pigmentation. The minimum quorum sensing inhibitory concentration (MQSIC) was defined as the lowest compound’s concentration allowing bacterial growth (shown by turbidity) without the visible production of purple pigmentation.

#### Quantification of violacein.

The inhibitory effect of selected compounds (**4a**, **4b** and **4c**) on violacein production was further quantified using a spectrophotometric method. After collection of MIC and MQSIC data, the plates were centrifuged at 4000 rpm for 20 min, and the supernatant was discarded. Then, the bacterial pellet was resuspended in 1 mL of DMSO, and the plates were further left in an orbital shaker for 10–15 min. Then, 200 µL of the supernatant was transferred into a 96-well microplate, and the optical density was measured at 595 nm. The percentage of violacein inhibition was calculated using the following formula:


$$\%\;violacein\;inhibition\;=\;100\;-\;[({OD}_{sample\;}-\;{OD}_{blank\;})/({OD}_{control\;}-\;{OD}_{blamk\;})\;\times \;100].$$

The IC_50_ values of the test compounds were defined as the concentrations inhibiting 50% of violacein production. These values were calculated by plotting the percentages of violacein production versus the concentrations using GraphPad Prism software. Samples were tested in triplicate, and each experiment was repeated three times.

### Kinetics of bacterial growth and biofilm formation at sub-MIC concentrations

The effect of sub-MIC concentrations on bacterial growth and biofilm was evaluated by performing the kinetics of bacterial growth and biofilm formation at MIC, 1/2 xMIC, 1/4 xMIC, 1/8 xMIC, 1/16 xMIC and 1/32xMIC. Then, the average of optical density values obtained at 570 nm, were plotted against the concentrations.

### Statistical analysis

The data are presented as the mean ± standard deviation (SD) of three independent experiments. Statistical differences between the IC_50_ values inhibiting the violacein production of samples and the reference compound (vanillin) were assessed by two-way ANOVA followed by Sidak’s multiple comparisons test in GraphPad Prism software.

## Results

### Chemistry

**Fig 1:**
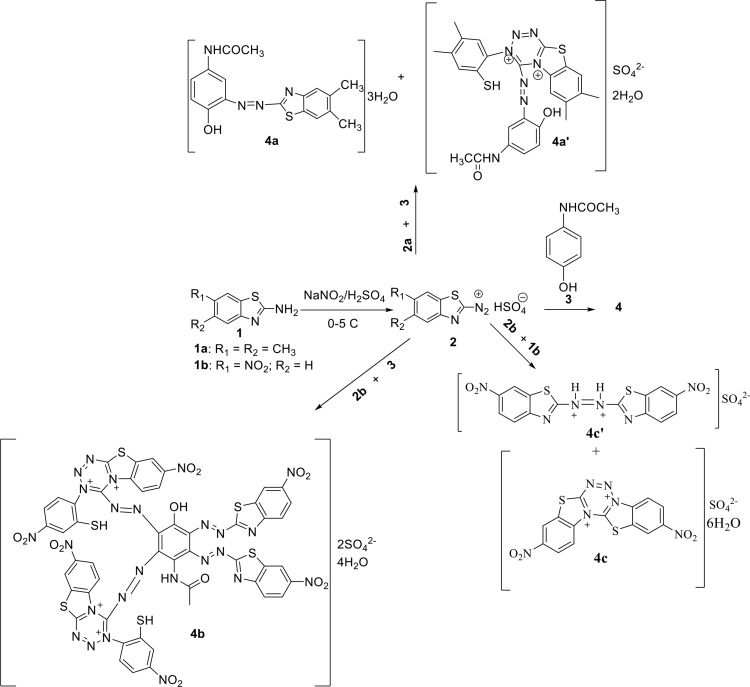
Reactions’ sequences to compounds 4.

The synthesis of 1,2,3,5-tetrazine and azo dyes **4** is shown in Fig 1. All synthesized compounds were synthesised according to previous experimental procedure [32]. The yields, the melting points and all the spectroscopic data for these compounds described in the present study are in full agreement with those originally reported [32].

### Assays for the inhibition of bacteria

Table 1 summarizes the minimum inhibitory concentrations (MICs) and minimum bactericidal concentrations (MBCs) of 1,2,3,5-tetrazine derivatives upon screening against *Klebsiella aerogenes*, *Acinetobacter baumannii*, *Enterococcus faecium* and *Staphylococcus epidermidis*. The incubation of test compounds with the four pathogens afforded MIC and MBC values ranging from 8 to 256 µg/mL and from 64 to 256 µg/mL, respectively (Table 1). Against *Klebsiella aerogenes*, *Acinetobacter baumannii*, and *Enterococcus faecium*, the most promising compound *viz.* **4b** showed bactericidal trend as evidenced by the ratios MBC/MIC (128/64, 64/16 and 32/8** **= **4**), which is more than 2. Also, compound **4a** (MBC/MIC: 128/16 = **8**) and **4c** (MBC/MIC: 64/16 = **4**) were found to be bactericidal when incubated with *Staphylococcus epidermidis*. Gentamicin, the standard antibiotic agent showed antibacterial activity against the tested bacteria with MIC values ranging from 0.25 to 2 µg/mL.

**Table 1:** The minimum inhibitory concentrations (µg/mL) and the minimum bactericidal concentrations (µg/mL) of synthesized 1,2,3,5-tetrazine derivatives.

Samples	Bacterial strains							
	*Ka*		*Ab*		*Ef*		*Se*	
	MIC	MBC	MIC	MBC	MIC	MBC	MIC	MBC
**1a**	–	–	–	–	256	256	256	–
**1b**	256	–	256	–	128	256	128	256
**4a**	128	128	**64**	128	**32**	**64**	**16**	128
**4a’**	256	–	–	–	128	256	128	256
**4b**	**64**	128	**16**	**64**	**8**	**32**	**32**	**64**
**4c**	128	256	**32**	128	**32**	128	**16**	**64**
**4c’**	256	–	128	–	–	–	256	–
Gentamicin	2	4	1	2	0.5	2	0.25	1

– = > 256 µg/mL *Ka: Klebsiella aerogenes, Ab: Acinetobacter baumannii, Ef: Enterococcus faecium, Se: Staphylococcus epidermidis*.

### Anti-biofilm activity

The anti-biofilm effect of 1,2,3,5-tetrazine derivatives was evaluated on biofilms formed by *Klebsiella aerogenes*, *Acinetobacter baumannii*, *Enterococcus faecium*, and *Staphylococcus epidermidis*. The percentage of biofilm formation and eradication are summarized in Table 2. The degree of anti-biofilm activity of the test compounds was classified as highly and poorly actives for percentages of inhibition of >50%, and 0 < % < 50, respectively. Any compound with inhibition percentage of antibiofilm formation of 0 was considered inactive [36]. Compounds **4a** (52–86.5%), **4b** (57.7–79.4%) and **4c** (59.9–80.3%) prevented biofilm formation in all the bacteria tested with percentages of inhibition >50%.

**Table 2:** Percentages of inhibition of biofilm formation and eradication of synthesized 1,2,3,5-tetrazine derivatives.

Samples	Biofilm formation inhibition (%)				Biofilm eradication (%)			
	*Ka*	*Ab*	*Ef*	*Se*	*Ka*	*Ab*	*Ef*	*Se*
**1a**	4.8 ± 0.5	8.5 ± 1.2	18.9 ± 0.6	15.7 ± 1.7	−6.4 ± 1.3	3.5 ± 0.2	3.5 ± 0.1	12.5 ± 1.5
**1b**	13.7 ± 0.9	15.7 ± 1.4	25.3 ± 0.4	35.2 ± 2.7	5.6 ± 0.6	5.0 ± 1.2	5.4 ± 0.3	24.3 ± 2.7
**4a**	68.5 ± 5.2	86.5 ± 5.2	56.6 ± 3.4	52.0 ± 3.5	52.4 ± 4.6	75.4 ± 4.7	34.5 ± 2.4	42.0 ± 4.3
**4a’**	28.9 ± 3.4	4.3 ± 0.4	37.4 ± 3.8	28.1 ± 1.6	13.5 ± 1.8	4.4 ± 1.0	0.09 ± 0.0	20.1 ± 2.6
**4b**	65.8 ± 6.9	79.4 ± 5.8	57.7 ± 2.4	70.3 ± 4.6	55.9 ± 3.6	66.3 ± 5.2	45.8 ± 2.4	61.0 ± 4.2
**4c**	65.8 ± 5.0	80.3 ± 8.7	59.9 ± 4.1	63.0 ± 4.2	59.0 ± 4.8	65.3 ± 5.7	48.0 ± 3.5	53.0 ± 5.8
**4c’**	32.0 ± 2.5	22.5 ± 2.6	9.0 ± 0.2	21.6 ± 1.8	15.9 ± 1.4	10.0 ± 2.1	−5.4 ± 0.7	19.0 ± 1.2
**Gentamicin**	95.7 ± 6.2	99.1 ± 7.9	98.0 ± 8.0	99.4 ± 5.1	84.7 ± 6.5	75.2 ± 6.4	72.2 ± 8.5	86.2 ± 8.5

***Ka*** *Klebsiella aerogenes,* ***Ab:*** *Acinetobacter baumannii* ***Ef:*** *Enterococcus faecium*, ***Se:*** *Staphylococcus epidermidis.*

Similarly, compounds **4b** (55.9–61.0%) and **4c** (53.0–65.3%) eradicated biofilms formed by *K. aerogenes*, *A. baumannii* and *S. epidermidis* with percentages of eradication of >50%. Moreover, compound **4a** eradicated biofilms formed by *K. aerogenes* and *A. baumannii* with percentages of inhibition of 52.4 and 75.4%, respectively. Gentamicin, the antibiotic that was used as a positive control showed percentages of inhibition for biofilm formation and eradication ranging from 95.7 and 99.4% and from 72.2 and 86.2%, respectively (Table 2).

Table 3 summarizes the minimum biofilm inhibitory concentrations (MBIC_50_s) and minimum biofilm eradicating concentrations (MBEC_50_s) of compounds (**4a**, **4b** and **4c**) that exhibited more than 50% inhibition upon anti-biofilm formation and eradication assays. The minimum biofilm inhibitory concentrations (MBIC_50_s) ranged from 5.29 to 54.91 µg/mL, 7.79 to 45.23 µg/mL, and 8.56 to 87.35 µg/mL for compounds **4a**, **4b** and **4c**, respectively (Table 3). Moreover, compounds **4a**, **4b** and **4c** displayed minimum biofilm eradication concentrations (MBIC_50_s) of 98.62 and 60.98 µg/mL, 54.21 and 15.70 µg/mL, as well as 105.08 and 29.55 µg/mL, respectively, when tested against *Klebsiella aerogenes* and *Acinetobacter baumannii* (Table 3). All three compounds showed minimal growth inhibition at sub-MIC concentrations (≤64 μg/mL), with optical density (OD) curves closely paralleling the untreated control. Notably, **4b** demonstrated the most pronounced separation between biofilm inhibition and growth curves, maintaining >80% biofilm reduction while showing <10% growth impact at 32 μg/mL (Supporting data S1 Fig). Similarly, the three compounds showed concentration-dependent suppression of violacein production, with **4b** exhibiting the steepest dose-response curve, achieving 50% inhibition at as low as 28.56 μg/mL concentration while permitting 90% of *Chromobacterium violaceum* growth (Supporting data S2 Fig).

**Table 3:** MBIC50 and MBEC50 values (µg/mL) of synthesized 1,2,3,5-tetrazine derivatives against bacteria pathogen strains.

Samples	MBIC_50_ (µg/mL)			
	*Ka*	*Ab*	*Ef*	*Se*
**4a**	54.91 ± 5.3	44.34 ± 4.1	31.87 ± 2.8	5.29 ± 1.1
**4b**	45.23 ± 4.7	6.54 ± 1.0	7.79 ± 1.5	19.22 ± 4.8
**4c**	87.35 ± 7.7	18.33 ± 2.5	30.51 ± 3.1	8.56 ± 1.3
Gentamicin	1.22 ± 0.1	2.28 ± 0.4	1.03 ± 0.2	0.11 ± 0.0
	**MBEC**_**50**_ **(µg/mL)**			
**4a**	98.62 ± 8.5	60.98 ± 5.4	nd	nd
**4b**	54.21 ± 4.9	15.70 ± 1.9	nd	25.44 ± 2.2
**4c**	105.08 ± 9.4	29.55 ± 3.1	nd	10.45 ± 2.1
Gentamicin	1.92 ± 0.1	0.95 ± 0.0	1.75 ± 0.2	0.18 ± 0.0

- = > 1024 µg/mL, *Ka: Klebsiella aerogenes, Ab: Acinetobacter baumannii Ef: Enterococcus faecium*, *Se: Staphylococcus epidermidis.*

### Assay for the inhibition of quorum sensing

#### Inhibition of violacein formation.

**Fig 2:**
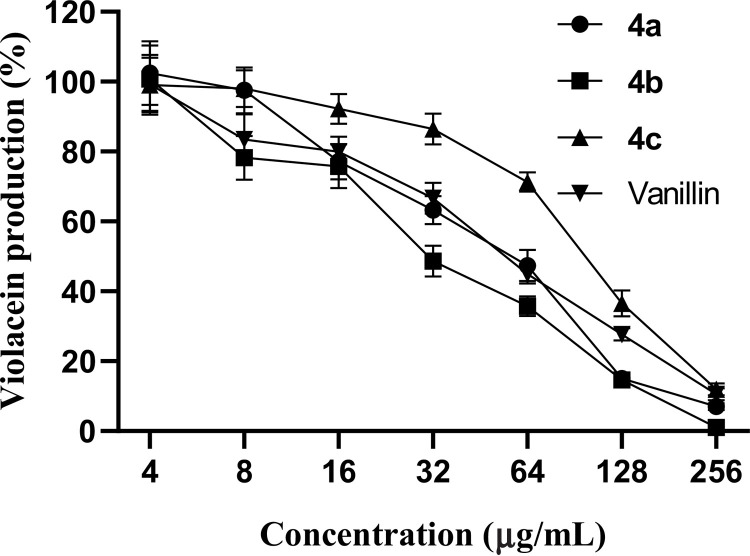
Percentage of violacein production in *Chromobacterium violaceum* by the most active synthesized 1,2,3,5-tetrazine derivatives (4a, 4b and 4c).

An indicator strain of bacteria, i.e., *Chromobacterium violaceum* 12472 was used to test the inhibitory potential of the 1,2,3,5-tetrazine derivatives (**4a**, **4b** and **4c**) on the production of violacein, a water soluble pigment that result from the disruption of quorum-sensing signals or inhibition of cell growth in a number of gram negative bacteria [37,38]. Herein, the inhibitory effects of compounds **4a**, **4b** and **4c** vis-à-vis violacein formation was evaluated at concentrations ranging from 0 to 256 µg/mL and the results are illustrated in Fig 2. As much as 256 µg/mL of compounds **4a**, **4b** and **4c** completely inhibited violacein production as evidenced by the trends of the curves that almost overlapped with the x axis at 256 µg/mL concentration. 50% inhibition of violacein formation was observed at approximately 32, 64 and 110 µg/mL concentrations for compounds **4a**, **4b** and **4c**, respectively, vs vanillin (50% inhibition of violacein formation at 64 µg/mL). These results demonstrate that compounds **4a**, **4b** and **4c** possess anti-quorum sensing activity.

#### Determination of the minimum inhibitory concentration (MIC) and minimum quorum sensing inhibitory concentration (MQSIC).

Table 4 summarizes the minimum inhibitory concentration (MIC) and minimum quorum sensing inhibitory concentration (MQSIC) of compounds **4a**, **4b** and **4c**, the most promising antibacterial tetrazine derivatives. Compounds **4a**, **4b** and **4c** revealed MIC and MQSIC values ranging from 256 to 512 µg/mL and from 64 and 256 µg/mL, respectively (Table 4). The concentrations of **4a**, **4b** and **4c** that inhibited 50% of violacein production were found to be 62.71, 28.56 and 107.45 µg/mL, respectively, vs vanillin (IC_50_: 56.75 µg/mL).

**Table 4:** Minimum quorum sensing inhibitory concentrations (MQSICs), minimum inhibitory concentrations (MICs), and concentrations inhibiting 50% of violacein production (IC50s) of compounds 4a, 4b and 4c.

Samples	MIC	MQSIC	IC_50_
**4a**	512	256	62.71 ± 5.11
**4b**	256	64	28.56 ± 1.24***
**4c**	256	128	107.45 ± 8.56***
**Vanillin**	256	128	56.75 ± 4.32

Statistical analysis was performed with Sidak’s multiple comparisons test using two-way ANOVA; ***p < 0.001.

## Discussion

Combating antimicrobial resistance (AMR) has been declared as a priority concern by the World Health Organization (WHO) ever since antibacterial AMR was directly responsible for 1.27 million global deaths in 2019 [4]. Adequate research and development on quality diagnosis, effective treatment of bacterial infections, and innovation are among the strategies that can slow down and eliminate bacterial drug resistance. To contribute toward the identification of effective treatments against drug resistant bacterial infections, numerous scientists have investigated the inhibitory effects of natural and synthetic compounds against different pathogens. This observation is exemplified by the number of recently published review articles on the antibacterial activity of heterocyclic compounds, including benzothiazole derivatives [39–41], tetrazine compounds [42,43], among others. On the other hand, the implication of biofilm formation [6,44,45] and quorum sensing system [46–48] in developing bacterial drug-resistance is prominent [49,50]. Thus, the search for effective antibacterial treatments with potential inhibition of biofilm formation and quorum sensing activity is valuable and might significantly contribute to antimicrobial drug discovery against multi-resistant bacteria. In point of fact, a number of antibacterial chemotypes were reported to exhibit anti-quorum sensing [51,52] and anti-biofilm activity [53,54]. However, the identification of antibacterial pharmacophores with novel features is still needed since none of these promising hit compounds is indicated to have entered the clinical trial phase, perhaps the reason being their unfavourable pharmacokinetic characteristics, their safety limits, and so on. As the benzothiazole and tetrazine rings hold promise as antibacterial potential candidates and that previously prepared derivatives from compounds bearing both the pharmacophores revealed non cytotoxicity against normal and cancer cells, the present study evaluated the antibacterial efficacy of 1,2,3,5-tetrazine amalgamated benzothiazole derivatives.

As a result, the synthesized 1,2,3,5-tetrazine derivatives showed MIC and MBC values ranging from 8 to 256 µg/mL and from 64 to 256 µg/mL, respectively, thus highlighting the antibacterial activity of these compounds. Compound **4b** was found to be the most potent, followed by compound **4c** and **4a**. Noteworthy, compound **4b** harbours more benzothiazole rings than its counterparts **4a** and **4c**, thus justifying the high inhibition of selected bacteria by compound **4b**.

Similarly, the compound **4a** contain a number of nitro groups, which might have aided in the observed antibacterial activity. By contrast, compound **4c** bears only two nitro groups, whereas compound **4a** do not contain any nitro moiety. The chemical and physical properties of the nitro group (−NO_2_) including its size, electron-withdrawing ability, polarity, ability to form hydrogen bonds and redox properties contribute to its key role in the action of many drugs, especially antimicrobial agents [55]. Accumulated evidence has shown the significant role of the nitro group in the inhibition of several bacteria [56–58].

The anti-biofilm and anti-quorum sensing effects of the most promising derivatives were also evaluated. As a result, compounds **4a**, **4b** and **4c**, inhibited biofilm formation with minimum biofilm inhibitory concentrations (MBIC_50_s) ranging from 5.29 to 54.91 µg/mL, 7.79 to 45.23 µg/mL, and 8.56 to 87.35 µg/mL for compounds **4a**, **4b** and **4c**, respectively. Moreover, compounds **4a**, **4b** and **4c** displayed minimum biofilm eradication concentrations (MBIC_50_s) of 98.62 and 60.98 µg/mL, 54.21 and 15.70 µg/mL, as well as 105.08 and 29.55 µg/mL, respectively, when tested against *Klebsiella aerogenes* and *Acinetobacter baumannii.* As shown in the supporting information (S1 and S2 Fig), the sub-inhibitory concentrations of compounds **4a**, **4b** and **4c** did not significantly affect the bacterial growth, biofilm formation and biological synthesis of violacein over the tested time period, thus justifying the selective inhibitory effects of the test compounds on these specific bacterial processes rather than a general decrease in the bacterial cell viability. These results are consistent with previously reported data on the anti-quorum sensing effects of benzothiazole derivatives containing an isopropanolamine moiety [51], pyrazole-based benzothiazoles [59], etc. Moreover, series of 2-azidobenzothiazoles [51] and benzothiazole‒urea hybrids [60] were reported to exhibit anti-biofilm activity. On the other hand, tetrazine groups are reputed for their antibacterial potential [61,62]. In general, the antibacterial activity of benzothiazole derivatives has been attributed to binding onto enzymes that are important for essential processes in the bacterial cells, such as cell-wall synthesis, cell division, and DNA replication [63,64]. Inhibition of violacein formation has been used as an approach to determine anti-quorum sensing activity [65]. The antibacterial, anti-biofilm, and anti-quorum sensing (QS) activities of the synthesized 1,2,3,5-tetrazine-benzothiazole hybrids (**4a-4c**) can be attributed to their unique structural features and potential interactions with bacterial targets. The potent bactericidal effects of **4b** (MIC: 8–64 µg/mL) likely stem from the benzothiazole moieties. These heterocycles may inhibit DNA gyrase or topoisomerase IV, which is a critical enzyme for bacterial DNA replication [63]. The additional benzothiazole rings in **4b** could improve target binding affinity compared to **4a** and **4c**. At sub-MIC concentrations, **4a-4c** selectively inhibited biofilm formation and violacein production without affecting bacterial growth. These results suggest the disruption of extracellular polymeric substances (EPS), since **t**he azo and tetrazine groups are well known to interfere with EPS synthesis [66]. Growing evidence has shown that QS interference by **v**iolacein suppression might result from the inhibition of the CviR/I system in *C. violaceum* [65,67]. The planar structure of compound **4b** may competitively bind LuxR-type receptors, thus blocking the signal transduction [51].

## Conclusions

This work has unveiled the antibacterial, anti-quorum sensing and anti-biofilm formation of selected 1,2,3,5-tetrazine-benzothiazole hybrids, which can be prospected as potential pharmacophores for the discovery of effective antibacterial agents. Future studies could dissect the mechanisms of action of the antibacterial compounds using transcriptional analysis such as RT-qPCR of QS genes, or genetic knockouts to confirm target specificity. Nevertheless, our findings contribute to the rationale for developing sub-MIC therapies targeting virulence, particularly in biofilm-associated infections where conventional antibiotics have failed due to several factors, including the physical barrier of the biofilm matrix, slow bacterial growth rates, and the presence of persistent cells within the biofilm.

## Supporting information

S1 Fig Effect of selected compounds at sub-inhibitory concentrations on biofilm and bacterial growth inhibition. **A:** effect of compound **4a**, **B:** effect of compound **4b**, **C:** effect of compound **4c**, **MIC:** minimum inhibitory concentration.
(DOCX)

S2 Fig Effect of selected compounds 4a, 4b and4c on*Chromobacterium violaceum* growth at sub-inhibitory concentrations. **MIC:** minimum inhibitory concentration.
(DOCX)

## References

[1] O’Neill L, Manzanilla EG, Ekhlas D, Leonard FC. Antimicrobial Resistance in Commensal Escherichia coli of the Porcine Gastrointestinal Tract. Antibiotics (Basel). 2023;12(11):1616. doi: 10.3390/antibiotics12111616 37998818 PMC10669415

[2] Oliveira M, Antunes W, Mota S, Madureira-Carvalho Á, Dinis-Oliveira RJ, Dias da Silva D. An Overview of the Recent Advances in Antimicrobial Resistance. Microorganisms. 2024;12(9):1920. doi: 10.3390/microorganisms12091920 39338594 PMC11434382

[3] Antimicrobial Resistance Collaborators. Global burden of bacterial antimicrobial resistance in 2019: a systematic analysis. Lancet. 2022;399(10325):629–55. doi: 10.1016/S0140-6736(21)02724-0 35065702 PMC8841637

[4] World Health Organization (WHO). The Fact Sheets. Antimicrobial Resistance. 2024. [cited 09th October 2024] Available from: https://www.who.int/news-room/fact-sheets/detail/antimicrobial-resistance

[5] Rajput A, Thakur A, Sharma S, Kumar M. aBiofilm: a resource of anti-biofilm agents and their potential implications in targeting antibiotic drug resistance. Nucleic Acids Res. 2018;46(D1):D894–900. doi: 10.1093/nar/gkx1157 29156005 PMC5753393

[6] Sharma S, Mohler J, Mahajan SD, Schwartz SA, Bruggemann L, Aalinkeel R. Microbial Biofilm: A Review on Formation, Infection, Antibiotic Resistance, Control Measures, and Innovative Treatment. Microorganisms. 2023;11(6):1614. doi: 10.3390/microorganisms11061614 37375116 PMC10305407

[7] Juszczuk-Kubiak E. Molecular Aspects of the Functioning of Pathogenic Bacteria Biofilm Based on Quorum Sensing (QS) Signal-Response System and Innovative Non-Antibiotic Strategies for Their Elimination. Int J Mol Sci. 2024;25(5):2655. doi: 10.3390/ijms25052655 38473900 PMC10931677

[8] Singh S, Singh SK, Chowdhury I, Singh R. Understanding the Mechanism of Bacterial Biofilms Resistance to Antimicrobial Agents. Open Microbiol J. 2017;11:53–62. doi: 10.2174/1874285801711010053 28553416 PMC5427689

[9] Abebe GM. The Role of Bacterial Biofilm in Antibiotic Resistance and Food Contamination. Int J Microbiol. 2020;2020:1705814. doi: 10.1155/2020/1705814 32908520 PMC7468660

[10] Shree P, Singh CK, Sodhi KK, Surya JN, Singh DK. Biofilms: Understanding the structure and contribution towards bacterial resistance in antibiotics. Medicine in Microecology. 2023;16:100084. doi: 10.1016/j.medmic.2023.100084

[11] Zhao X, Yu Z, Ding T. Quorum-Sensing Regulation of Antimicrobial Resistance in Bacteria. Microorganisms. 2020;8(3):425. doi: 10.3390/microorganisms8030425 32192182 PMC7143945

[12] Kirisits MJ, Parsek MR. Does Pseudomonas aeruginosa use intercellular signalling to build biofilm communities?. Cell Microbiol. 2006;8(12):1841–9. doi: 10.1111/j.1462-5822.2006.00817.x 17026480

[13] Spoering AL, Gilmore MS. Quorum sensing and DNA release in bacterial biofilms. Curr Opin Microbiol. 2006;9(2):133–7. doi: 10.1016/j.mib.2006.02.004 16529982

[14] Hooshangi S, Bentley WE. From unicellular properties to multicellular behavior: bacteria quorum sensing circuitry and applications. Curr Opin Biotechnol. 2008;19(6):550–5. doi: 10.1016/j.copbio.2008.10.007 18977301

[15] Wu L, Luo Y. Bacterial Quorum-Sensing Systems and Their Role in Intestinal Bacteria-Host Crosstalk. Front Microbiol. 2021;12:611413. doi: 10.3389/fmicb.2021.611413 33584614 PMC7876071

[16] Zhang A, Chu W-H. Anti-Quorum Sensing Activity of Forsythia suspense on Chromobacterium violaceum and Pseudomonas aeruginosa. Pharmacogn Mag. 2017;13(50):321–5. doi: 10.4103/0973-1296.204547 28539728 PMC5421433

[17] Sadik A, Viswaswar JP, Rajamoney A, Rekha A, Raj DM, Prakashan D, et al. Mitigation of quorum sensing mediated virulence factors of Pseudomonas aeruginosa: the role of Meldrum’s acid activated furan. Front Microbiol. 2024;14:1272240. doi: 10.3389/fmicb.2023.1272240 38235424 PMC10791761

[18] Sionov RV, Steinberg D. Targeting the Holy Triangle of Quorum Sensing, Biofilm Formation, and Antibiotic Resistance in Pathogenic Bacteria. Microorganisms. 2022;10(6):1239. doi: 10.3390/microorganisms10061239 35744757 PMC9228545

[19] Grooters KE, Ku JC, Richter DM, Krinock MJ, Minor A, Li P, et al. Strategies for combating antibiotic resistance in bacterial biofilms. Front Cell Infect Microbiol. 2024;14:1352273. doi: 10.3389/fcimb.2024.1352273 38322672 PMC10846525

[20] Kalia VC. Quorum sensing inhibitors: an overview. Biotechnol Adv. 2013;31(2):224–45. doi: 10.1016/j.biotechadv.2012.10.004 23142623

[21] Kalia VC, Patel SKS, Lee J-K. Bacterial biofilm inhibitors: An overview. Ecotoxicol Environ Saf. 2023;264:115389. doi: 10.1016/j.ecoenv.2023.115389 37634478

[22] Asfour HZ. Anti-Quorum Sensing Natural Compounds. J Microsc Ultrastruct. 2018;6(1):1–10. doi: 10.4103/JMAU.JMAU_10_18 30023261 PMC6014249

[23] Bouyahya A, Chamkhi I, Balahbib A, Rebezov M, Shariati MA, Wilairatana P, et al. Mechanisms, Anti-Quorum-Sensing Actions, and Clinical Trials of Medicinal Plant Bioactive Compounds against Bacteria: A Comprehensive Review. Molecules. 2022;27(5):1484. doi: 10.3390/molecules27051484 35268585 PMC8911727

[24] Iaconis A, De Plano LM, Caccamo A, Franco D, Conoci S. Anti-Biofilm Strategies: A Focused Review on Innovative Approaches. Microorganisms. 2024;12(4):639. doi: 10.3390/microorganisms12040639 38674584 PMC11052202

[25] Booysen IN, Ismail MB, Akerman MP. N-[(E)-Thio-phen-2-yl-methyl-idene]-1,3-benzothia-zol-2-amine. Acta Crystallogr Sect E Struct Rep Online. 2012;68(Pt 8):o2489. doi: 10.1107/S1600536812030498 22904931 PMC3414944

[26] Sumit, Kumar A, Mishra AK. Advancement in Pharmacological Activities of Benzothiazole and its Derivatives: An Up to Date Review. Mini Rev Med Chem. 2021;21(3):314–35. doi: 10.2174/1389557520666200820133252 32819243

[27] Zhilitskaya LV, Shainyan BA, Yarosh NO. Modern Approaches to the Synthesis and Transformations of Practically Valuable Benzothiazole Derivatives. Molecules. 2021;26(8):2190. doi: 10.3390/molecules26082190 33920281 PMC8070523

[28] Catalano A, Rosato A, Salvagno L, Iacopetta D, Ceramella J, Fracchiolla G, et al. Benzothiazole-Containing Analogues of Triclocarban with Potent Antibacterial Activity. Antibiotics (Basel). 2021;10(7):803. doi: 10.3390/antibiotics10070803 34356724 PMC8300675

[29] Yadav RK, Kumar R, Singh H, Mazumdar A, Chauhan B, et al. Recent Insights on Synthetic Methods and Pharmacological Potential in Relation with Structure of Benzothiazoles. Med Chem. 2023;19(4):325–60. doi: 10.2174/1573406418666220820110551 35993459

[30] El-Reedy AAM, Soliman NK. Synthesis, biological activity and molecular modeling study of novel 1,2,4-triazolo[4,3-b][1,2,4,5]tetrazines and 1,2,4-triazolo[4,3-b][1,2,4]triazines. Sci Rep. 2020;10(1):6137. doi: 10.1038/s41598-020-62977-x 32273529 PMC7145827

[31] Ishmetova RI, Ignatenko NK, Gerasimova NA, Belyaev DV, Butorin II, Konovalova OA. 3,6-disubstituted derivatives of 1,2,4,5-tetrazine with pyridinyl amidine moieties and condensed systems on their basis: synthesis, docking, and antibacterial activity. Russ Chem Bull. 2024;73:1686–97.

[32] Tsemeugne J, Bah YA, Dzoyem JP, Ndefongang JN, Famuyide IM, McGaw LJ, et al. Synthesis and anticancer activity evaluation of some new 1,2,3,5-tetrazine derivatives attached to benzothiazole moiety. Arkivoc. 2022; part ix:73–89.

[33] Bisso BN, Makuété AL, Tsopmene JU, Dzoyem JP. Biofilm Formation and Phospholipase and Proteinase Production in Cryptococcus neoformans Clinical Isolates and Susceptibility towards Some Bioactive Natural Products. ScientificWorldJournal. 2023;2023:6080489. doi: 10.1155/2023/6080489 37035538 PMC10081907

[34] Ahmad A, Viljoen AM, Chenia HY. The impact of plant volatiles on bacterial quorum sensing. Lett Appl Microbiol. 2015;60(1):8–19. doi: 10.1111/lam.12343 25346138

[35] Choo JH, Rukayadi Y, Hwang J-K. Inhibition of bacterial quorum sensing by vanilla extract. Lett Appl Microbiol. 2006;42(6):637–41. doi: 10.1111/j.1472-765X.2006.01928.x 16706905

[36] Sandasi M, Leonard C, Van Vuuren S, Viljoen A. Peppermint (Mentha piperita) inhibits microbial biofilms in vitro. S Afr J Bot. 2011;77(1):80–5.

[37] Kocak G, Tamfu A, Bütün V, Ceylan O. Synthesis of quaternary piperazine methacrylate homopolymers and their antibioflm and anti-quorum sensing efects on pathogenic bacteria. J Appl Polym Sci. 2021;138(21):50466.

[38] Tamfu AN, Kucukaydin S, Ceylan O, Sarac N, Duru EM. Phenolic composition, enzyme inhibitory and anti-quorum sensing activities of cinnamon (Cinnamomum zeylanicum blume) and basil (Ocimum basilicum linn). Chem Afr. 2021;4(4):759–67.

[39] Franchini C, Muraglia M, Corbo F, Florio MA, Di Mola A, Rosato A, et al. Synthesis and biological evaluation of 2-mercapto-1,3-benzothiazole derivatives with potential antimicrobial activity. Arch Pharm (Weinheim). 2009;342(10):605–13. doi: 10.1002/ardp.200900092 19753564

[40] Ali R, Siddiqui N. Biological Aspects of Emerging Benzothiazoles: A Short Review. Journal of Chemistry. 2013;2013(1). doi: 10.1155/2013/345198

[41] Kashyap P, Verma S, Gupta P, Narang R, Lal S, Devgun M. Recent insights into antibacterial potential of benzothiazole derivatives. Med Chem Res. 2023;:1–31. doi: 10.1007/s00044-023-03077-z 37362317 PMC10226039

[42] Neamah IJ, Baqer FM, Hassan BA. Review nomenclature systems of tetrazine and its pharmacology application. Int J Pharm Sci Res. 2022;7:1–6.

[43] Abdulridha MM, Hassan BA, Neamah IJ. Review theoretical study of synthesis and pharmaceutical study of tetrazine derivatives. Ann Rom Soc Cell Biol. 2022;26(01):1657–69.

[44] Rather MA, Gupta K, Mandal M. Microbial biofilm: formation, architecture, antibiotic resistance, and control strategies. Braz J Microbiol. 2021;52(4):1701–18. doi: 10.1007/s42770-021-00624-x 34558029 PMC8578483

[45] Mirghani R, Saba T, Khaliq H, Mitchell J, Do L, Chambi L, et al. Biofilms: Formation, drug resistance and alternatives to conventional approaches. AIMS Microbiol. 2022;8(3):239–77. doi: 10.3934/microbiol.2022019 36317001 PMC9576500

[46] Gupta DS, Kumar MS. The implications of quorum sensing inhibition in bacterial antibiotic resistance- with a special focus on aquaculture. J Microbiol Methods. 2022;203:106602. doi: 10.1016/j.mimet.2022.106602 36270462

[47] Patel R, Soni M, Soyantar B, Shivangi S, Sutariya S, Saraf M, et al. A clash of quorum sensing vs quorum sensing inhibitors: an overview and risk of resistance. Arch Microbiol. 2023;205(4):107. doi: 10.1007/s00203-023-03442-x 36881156

[48] Rodríguez-Urretavizcaya B, Vilaplana L, Marco M-P. Strategies for quorum sensing inhibition as a tool for controlling Pseudomonas aeruginosa infections. Int J Antimicrob Agents. 2024;64(5):107323. doi: 10.1016/j.ijantimicag.2024.107323 39242051

[49] Vashistha A, Sharma N, Nanaji Y, Kumar D, Singh G, Barnwal RP, et al. Quorum sensing inhibitors as Therapeutics: Bacterial biofilm inhibition. Bioorg Chem. 2023;136:106551. doi: 10.1016/j.bioorg.2023.106551 37094480

[50] Hemmati J, Nazari M, Abolhasani FS, Ahmadi A, Asghari B. In vitro investigation of relationship between quorum-sensing system genes, biofilm forming ability, and drug resistance in clinical isolates of Pseudomonas aeruginosa. BMC Microbiol. 2024;24(1):99. doi: 10.1186/s12866-024-03249-w 38528442 PMC10962089

[51] Chu P-L, Feng Y-M, Long Z-Q, Xiao W-L, Ji J, Zhou X, et al. Novel Benzothiazole Derivatives as Potential Anti-Quorum Sensing Agents for Managing Plant Bacterial Diseases: Synthesis, Antibacterial Activity Assessment, and SAR Study. J Agric Food Chem. 2023;71(17):6525–40. doi: 10.1021/acs.jafc.2c07810 37073686

[52] Talla RM, Tamfu AN, Wakeu BNK, Ceylan O, Mbazoa CD, Kapche GDWF, et al. Evaluation of anti-quorum sensing and antibiofilm effects of secondary metabolites from Gambeya lacourtiana (De Wild) Aubr. & Pellegr against selected pathogens. BMC Complement Med Ther. 2023;23(1):300. doi: 10.1186/s12906-023-04115-4 37620848 PMC10464238

[53] Stojković D, Petrović J, Carević T, Soković M, Liaras K. Synthetic and Semisynthetic Compounds as Antibacterials Targeting Virulence Traits in Resistant Strains: A Narrative Updated Review. Antibiotics (Basel). 2023;12(6):963. doi: 10.3390/antibiotics12060963 37370282 PMC10295040

[54] Adeyemo RO, Famuyide IM, Dzoyem JP, Lyndy Joy M. Anti-Biofilm, Antibacterial, and Anti-Quorum Sensing Activities of Selected South African Plants Traditionally Used to Treat Diarrhoea. Evid Based Complement Alternat Med. 2022;2022:1307801. doi: 10.1155/2022/1307801 36212949 PMC9534605

[55] Rice AM, Long Y, King SB. Nitroaromatic Antibiotics as Nitrogen Oxide Sources. Biomolecules. 2021;11(2):267. doi: 10.3390/biom11020267 33673069 PMC7918234

[56] Lobana TS, Indoria S, Sood H, Arora DS, Kaur M, Jasinski JP. Synthesis of (3-nitro-2-oxo-benzaldehyde thiosemicarbazonato)-zinc(II) complexes: the position of nitro group in phenyl ring alters antimicrobial activity against K. pneumoniae 1, S. typhimurium 2, MRSA and C. albicans. Dalton Trans. 2021;50(20):6823–33. doi: 10.1039/d1dt00657f 33890612

[57] Noriega S, Cardoso-Ortiz J, López-Luna A, Cuevas-Flores MDR, Flores De La Torre JA. The Diverse Biological Activity of Recently Synthesized Nitro Compounds. Pharmaceuticals (Basel). 2022;15(6):717. doi: 10.3390/ph15060717 35745635 PMC9230682

[58] Ayoup MS, Rabee AR, Abdel-Hamid H, Harras MF, El Menofy NG, Ismail MMF. Exploration of Nitroaromatic Antibiotics via Sanger’s Reagent: Synthesis, In Silico, and Antimicrobial Evaluation. ACS Omega. 2022;7(6):5254–63. doi: 10.1021/acsomega.1c06383 35187340 PMC8851660

[59] Gabr MT, El-Gohary NS, El-Bendary ER, El-Kerdawy MM, Ni N, Shaaban MI. Synthesis, antimicrobial, antiquorum-sensing and cytotoxic activities of new series of benzothiazole derivatives. Chin Chem Lett. 2015;26(12):1522–8.

[60] Zha L, Xie Y, Wu C, Lei M, Lu X, Tang W, et al. Novel benzothiazole‒urea hybrids: Design, synthesis and biological activity as potent anti-bacterial agents against MRSA. Eur J Med Chem. 2022;236:114333. doi: 10.1016/j.ejmech.2022.114333 35397402

[61] Shawali AS, Tawfik NM. Novel facile synthesis of imidazo[1,2-b]-[1,2,4,5]tetrazines with potential antimicrobial activity. Arch Pharmacal Res. 2009; 32: 975–82. 10.1007/s12272-009-1701-4 19641877

[62] Al-Omair MA, Sayed AR, Youssef MM. Synthesis of novel triazoles, tetrazine, thiadiazoles and their biological activities. Molecules. 2015;20(2):2591–610. doi: 10.3390/molecules20022591 25648599 PMC6272378

[63] Gjorgjieva M, Tomašič T, Kikelj D, Mašič LP. Benzothiazole-based Compounds in Antibacterial Drug Discovery. Curr Med Chem. 2018;25(38):5218–36. doi: 10.2174/0929867324666171009103327 28990510

[64] Haroun M. Review on the Developments of Benzothiazole-containing Antimicrobial Agents. Curr Top Med Chem. 2022;22(32):2630–59. doi: 10.2174/1568026623666221207161752 36503470

[65] Dimitrova PD, Ivanova V, Trendafilova A, Paunova-Krasteva T. Anti-Biofilm and Anti-Quorum-Sensing Activity of Inula Extracts: A Strategy for Modulating Chromobacterium violaceum Virulence Factors. Pharmaceuticals (Basel). 2024;17(5):573. doi: 10.3390/ph17050573 38794143 PMC11123807

[66] Roy R, Tiwari M, Donelli G, Tiwari V. Strategies for combating bacterial biofilms: A focus on anti-biofilm agents and their mechanisms of action. Virulence. 2018;9(1):522–54. doi: 10.1080/21505594.2017.1313372 28362216 PMC5955472

[67] Khan MA, Shahid M, Celik I, Khan HM, Shahzad A, Husain FM, et al. Attenuation of quorum sensing regulated virulence functions and biofilm of pathogenic bacteria by medicinal plant Artemisia annua and its phytoconstituent 1, 8-cineole. Microsc Res Tech. 2024;87(1):133–48. doi: 10.1002/jemt.24418 37728140

