# Supplementary material for: Antibacterial, antibiofilm and anti-quorum sensing activities of 1,2,3,5-tetrazine derivatives linked to a benzothiazole moiety
Source: PLoS One. 2025 Jun 3;20(6):e0318135. doi: 10.1371/journal.pone.0318135 (PMC12133013; doi:10.1371/journal.pone.0318135)
Supplement: Supplementary file 1 [file pone.0318135.s001.docx]

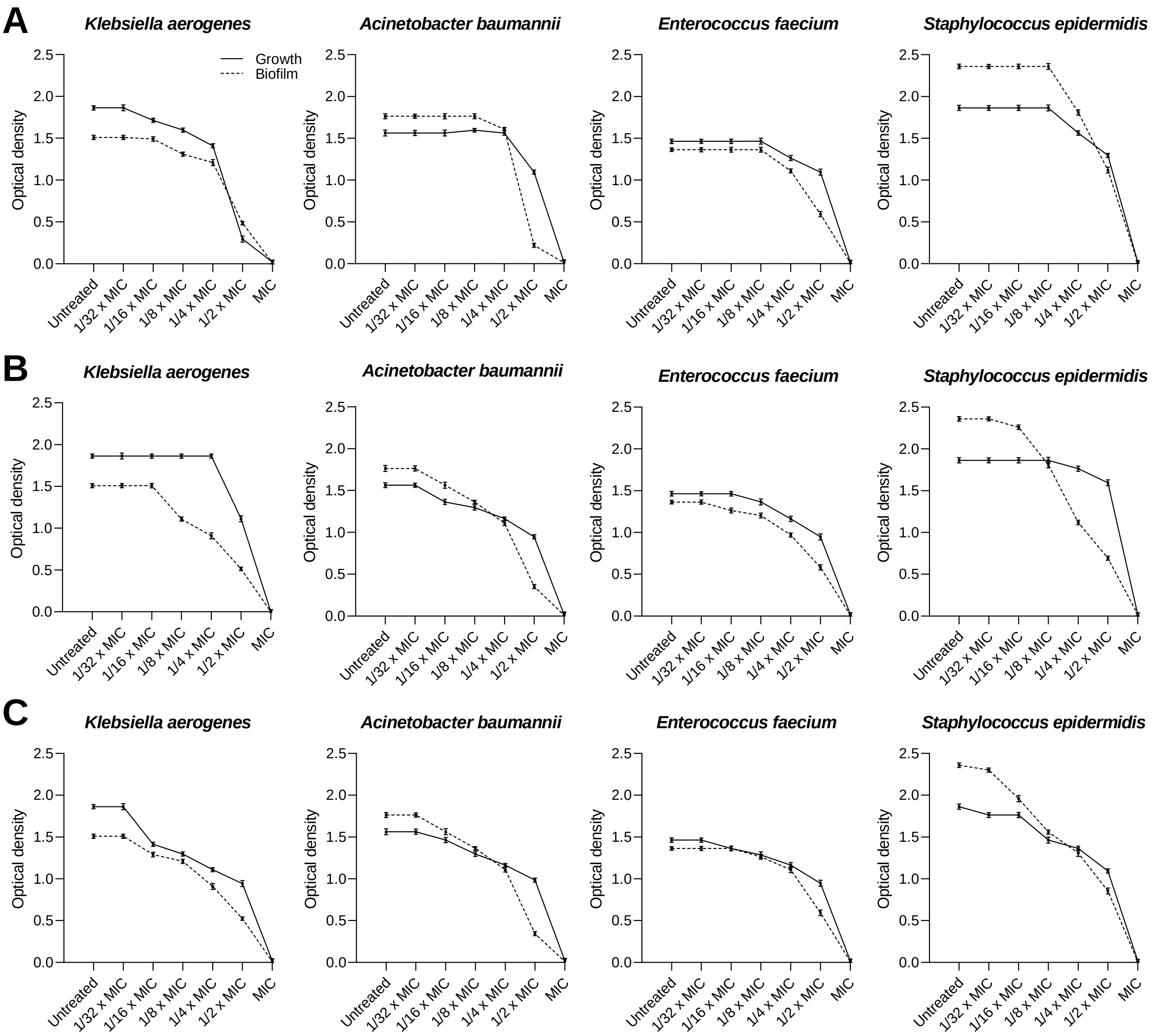
A
Klebsiella aerogenes
Acinetobacter baumannii
Enterococcus faecium
Staphylococcus epidermidis
Growth
Biofilm
Optical density
0.0
0.5
1.0
1.5
2.0
2.5
Untreated
1/32 x MIC
1/16 x MIC
1/8 x MIC
1/4 x MIC
1/2 x MIC
MIC
B
Klebsiella aerogenes
Acinetobacter baumannii
Enterococcus faecium
Staphylococcus epidermidis
C
Klebsiella aerogenes
Acinetobacter baumannii
Enterococcus faecium
Staphylococcus epidermidis
